# Rich stoichiometries of stable Ca-Bi system: Structure prediction and superconductivity

**DOI:** 10.1038/srep09326

**Published:** 2015-03-20

**Authors:** Xu Dong, Changzeng Fan

**Affiliations:** 1 State Key Laboratory of Metastable Materials Science and Technology, Yanshan University, Qinhuangdao 066004, China

## Abstract

Using a variable-composition *ab initio* evolutionary algorithm implemented in the USPEX code, we have performed a systematic search for stable compounds in the Ca-Bi system at different pressures. In addition to the well-known *tI*12-Ca_2_Bi and *oS*12-CaBi_2_, a few more structures were found by our calculations, among which phase transitions were also predicted in Ca_2_Bi (*tI*12 → *oI*12 → *hP*6), Ca_3_Bi_2_ (*hP*5 → *mC*20 → *aP*5) and CaBi (*tI*2 → *tI*8), as well as a new phase (Ca_3_Bi) with a *cF*4 structure. All the newly predicted structures can be both dynamically and thermodynamically stable with increasing pressure. The superconductive properties of *cF*4-CaBi_3_, *tI*2-CaBi and *cF*4-Ca_3_Bi were studied and the superconducting critical temperature *T_c_* can be as high as 5.16, 2.27 and 5.25 K, respectively. Different superconductivity behaviors with pressure increasing have been observed by further investigations.

Superconductivity has been deeply studied and developed very quickly since its discovery in 1911, but its origin remains enigmatic. The copper oxide family is of enduring interest for initiating energetic activities of high-temperature superconductivity^1^,^2^ and has been applied in a variety of fields. The discovery of the iron-based superconductors^3^, which is unconventional, has attracted great attention and aroused extensive research with the intention of finding new superconductors. Iron-based superconductors have been extended to various material groups, such as the so called 1111^3^,^4^, 122^5^, 111^6^, 11^7^ compounds, etc. Moreover, it is observed that compounds in which iron is completely substituted by other 3d, 4d, or 5d transition metals^8^,^9^, exhibit superconductivity. Since a large variety of phosphide, arsenide and antimonide superconductors have been found, attention is now focusing on bismuthides and related pnictide systems. So bismuth has been a part of various superconducting compounds, such as NiBi_3_^10^, Bi_4_O_4_S_3_^11^,^12^, CsBi_4_Te_6_^13^ and LaO_1-x_F_x_BiS_2_^14^. Recently, Sturza *et al*. reported a new complex alkaline earth intermetallic compound superconductor Ca_11_Bi_10-x_^15^ with *T_c_* ~ 2.2 K, which stimulates our interests to find new superconductors in the Ca-Bi system.

A large number of BCS superconductors have been theoretically proposed^16^,^17^,^18^ as the development of computational crystal structure prediction tools^19^,^20^ and methods. In this paper, we perform a systematic search for thermodynamically stable calcium bismuth at ambient and high pressure by using the variable-composition *ab initio* evolutionary algorithm^21^,^22^,^23^ and density functional theory (DFT). Here we predict several new structures at different pressures that were never reported, and discuss their structures, electronic structures and superconductivity properties of the selected structures.

## Results

### Crystal structure and structural properties of calcium bismuthides

Some reported experimental crystal structures in the Ca-Bi system are summarized by H. Kim *et*. *al*.^24^ and other similar Sr-Bi compounds have also been studied by first-principles calculations^25^. In this paper, we use the variable-composition evolutionary algorithm, which is very effective, to predict stable compositions and their structures. We have performed structure searches with up to 16 atoms in the unit cell at different pressures for the Ca-Bi system of all possible compositions.

**Figure 1:**
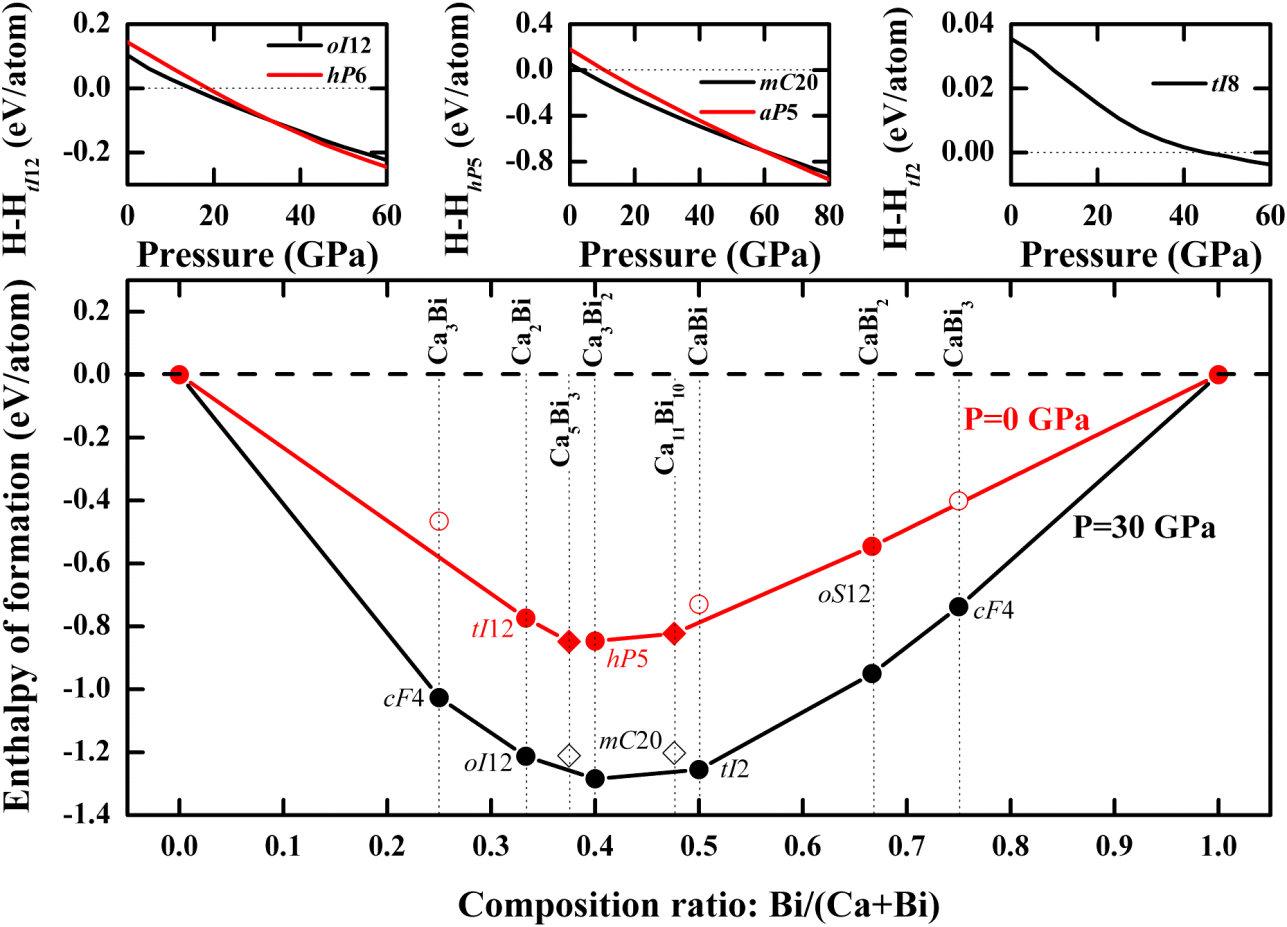
Enthalpy differences (top) and convex hull for the Ca-Bi system (bottom). Calculated enthalpy differences as a function of pressure relative to *tI*12 of Ca_2_Bi (top left), *hP*5 of Ca_3_Bi_2_ (top middle) and *tI*2 of CaBi (top right) and convex hull (bottom) for the Ca-Bi system at ambient pressure(red) and 30 GPa (black).

**Figure 2:**
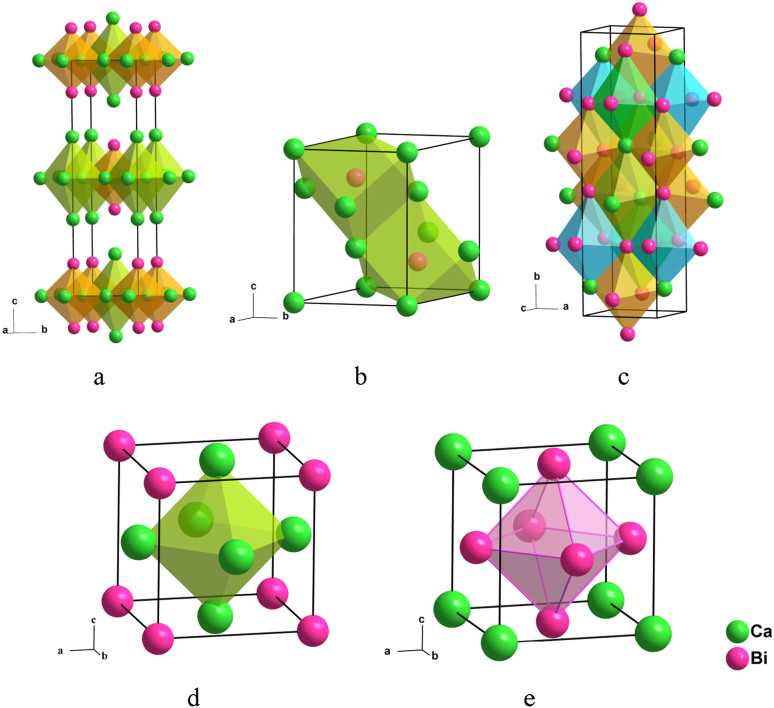
Crystal structures for (a): *tI*12-Ca_2_Bi, (b): *hP*5-Ca_3_Bi_2_, (c): *oS*12-CaBi_2_, (d): *cF*4-Ca_3_Bi and (e): *cF*4-CaBi_3_.

Fig. 1 shows the enthalpies of formation of the predicted structures. At ambient pressure, it can be clearly seen that there are three stable structures on the convex hull, i.e., *tI*12-Ca_2_Bi (Fig. 2a), *hP*5-Ca_3_Bi_2_ (Fig. 2b) and *oS*12-CaBi_2_ (Fig. 2c). The previously reported *oP*32-Ca_5_Bi_3_^26^ and *tI*84-Ca_11_Bi_10_^27^, although not found by our prediction possibly limited by the computational resources, are also on the convex hull, suggesting that they are thermodynamically stable. However, these two structures lie above the convex hull curve at 30 GPa, which implies that they will become metastable phases under high-pressure conditions.

**Figure 3:**
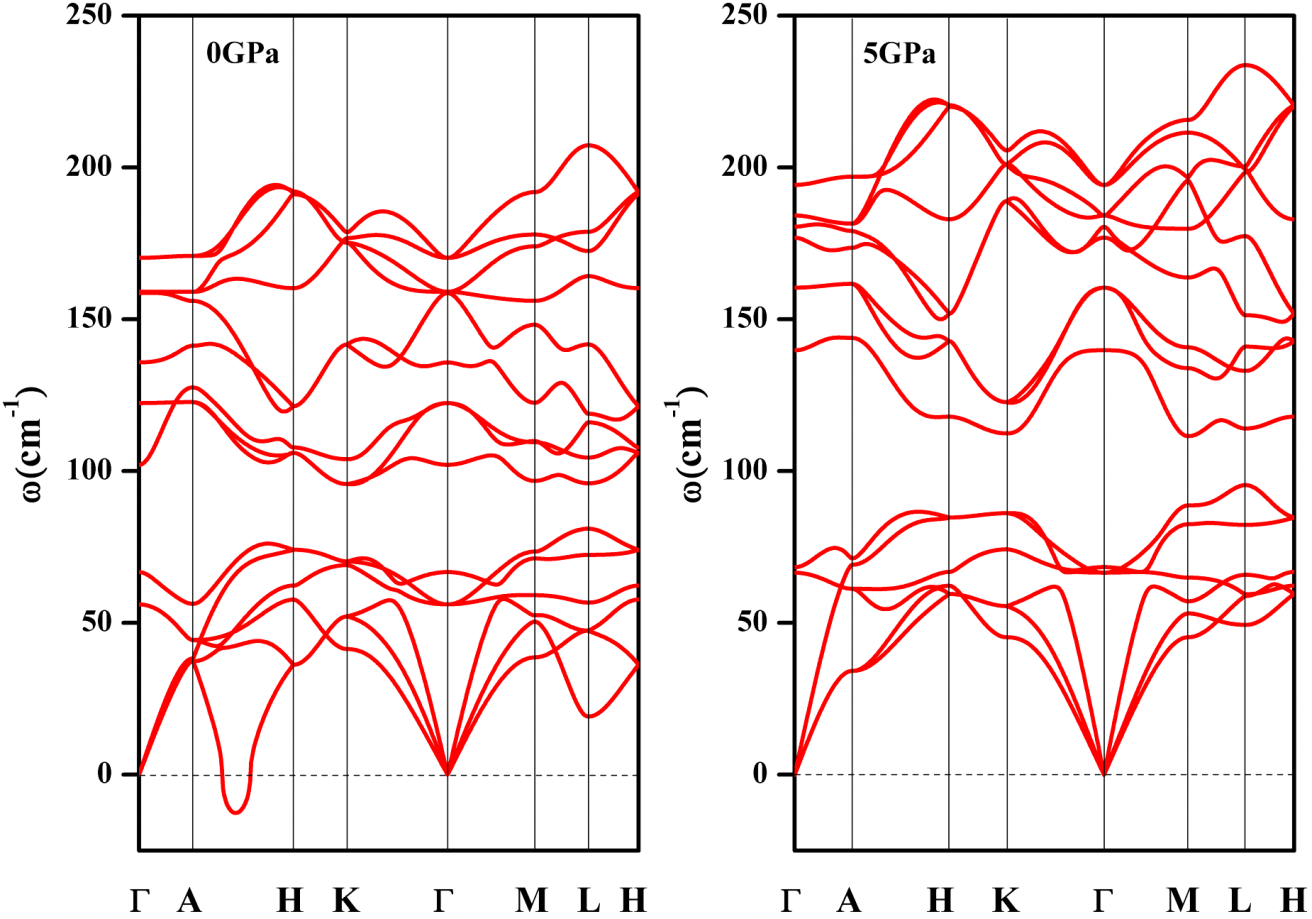
Phonon dispersion curves for the *hP*5-Ca_3_Bi_2_ at 0 GPa and 5 GPa.

The Ca_3_Bi_2_ phase has been reported in the literature without structural information. Our prediction indicates that it has a hexagonal structure of the La_2_O_3_ type, and belongs to the space group of 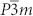 (164), with Pearson symbol *hP*5. Our calculations reveal that Ca_3_Bi_2_ undergoes a series phase transitions, *i*.*e*., from *hP*5 to *mC*20 (space group *C*2/*m*, 12) at 4 GPa and *mC*20 to *aP*5 (space group 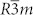 166) at about 59 GPa. The calculated phonon dispersion of *hP*5-Ca_3_Bi_2_ indicates that the phonon mode along the *A*-*H* direction is imaginary at 0 GPa, and it will be dynamically stable at high pressure, which can be seen in Fig. 3 (further study shows that the imaginary phonon mode along the *A*-*H* direction will disappear at above 0.5 ~ 0.6 GPa, which can be seen in Figure S1). We also observed a high-pressure phase transition in Ca_2_Bi, which is from *tI*12^28^ (space group *I*4/*mmm*, 139) to *oI*12 (space group *Cmmm*, 65) at 14 GPa and from *oI*12 to *hP*6 (space group *P*6_3_/*mmc*, 194) at 33GPa. For CaBi_2_, we predict an *oS*12 structure (space group *Cmcm*, 63) with lattice constants *a* = 4.782 Å, *b* = 17.160 Å, *c* = 4.598 Å at 0 GPa compared with the previously reported *a* = 4.701 Å, *b* = 17.053 Å, *c* = 4.613 Å^29^, which lies on the convex hull at ambient and high pressure, and is both dynamically and thermodynamically stable.

The previously reported Ca-Bi phase diagrams^30^ mentioned two phases, CaBi_3_ and CaBi, without structural information. This work suggests a cubic *cF*4 structure for CaBi_3_ (space group 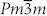, 221) and a tetragonal *tI*2 structure for CaBi (space group *P*4*/mmm*, 123), which are thermodynamically unstable at ambient pressure and can be stable at high pressure. The *cF*4-CaBi_3_ is very close to the convex hull curve but lies a little above it. In addition, another tetragonal *tI*8 structure of CaBi (space group *I*4_1_*/amd*, 141) was also predicted, which is proved to be thermodynamically more stable at above 45 GPa. Besides, our research also predicted a new phase of Ca-Bi system, *i*.*e*., *cF*4-Ca_3_Bi, the space group of which is the same with *cF*4-CaBi_3_, and can be thermodynamically stable at high pressure. In *cF*4-Ca_3_Bi, the Ca and Bi atoms occupy Wyckoff 3*c* and 1*a* positions, respectively. However, the situation of the occupancy is just the opposite in *cF*4-CaBi_3_, which can be seen in Figs. 2d and 2e. The structural parameters for the predicted structures are listed in Table 1.

### Electronic structures

**Figure 4:**
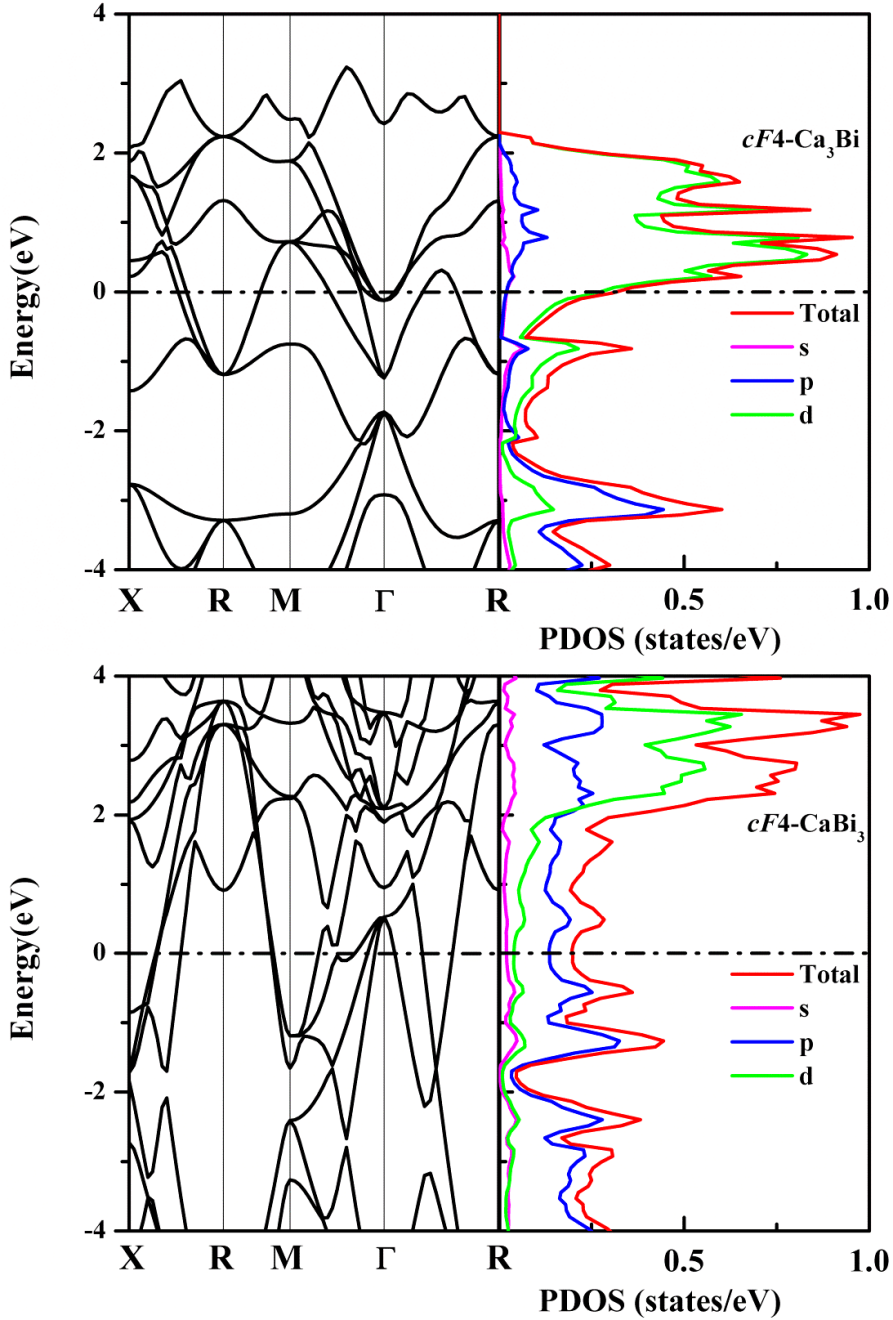
Band structure and partial density of states for *cF*4-Ca_3_Bi and *cF*4-CaBi_3_.

We calculated the band structures and density of states of all the predicted structures. All the calcium bismuthides are metallic except for the *hP*5-Ca_3_Bi_2_ structure, which is semiconductive at 0 GPa, with a narrow band gap of 0.42 eV and becomes metallic at around 20 GPa. Through the calculations we can conclude that the ratio of Ca and Bi will affect their contributions to the DOS at the Fermi level (*E_F_*). For example, if a calcium rich structure of Ca_x_Bi_1-x_, i.e., x > 0.5, the density of states at *E_F_* comes mainly from the calcium atoms, in particular Ca *d* states and bismuth *p* orbital contributes most to bismuth states. On the contrary, the density of states at *E_F_* comes mainly from the bismuth atoms in a bismuth rich structure, in particular Bi *p* states, and calcium *d* orbital contributes most to calcium states at the Fermi level. Fig. 4 shows the band structures and DOS of the *cF*4-Ca_3_Bi and *cF*4-CaBi_3_ structures. The density of states at *E_F_* of these two structures is 0.32 and 0.20 states/eV, respectively. A careful examination of the band structures show multiple steep bands crossing the Fermi level as well as flat bands, which is considered to be a necessary condition for superconductivity to occur^31^. As bismuth based materials may have strong spin-orbital coupling (SOC) effect^32^,^33^, the electronic structures of *cF*4-Ca_3_Bi and *cF*4-CaBi_3_ have been calculated by including the SOC effect and compared with those without SOC effect, as shown in Figure S2 and S3.

### Superconductivity properties

**Figure 5:**
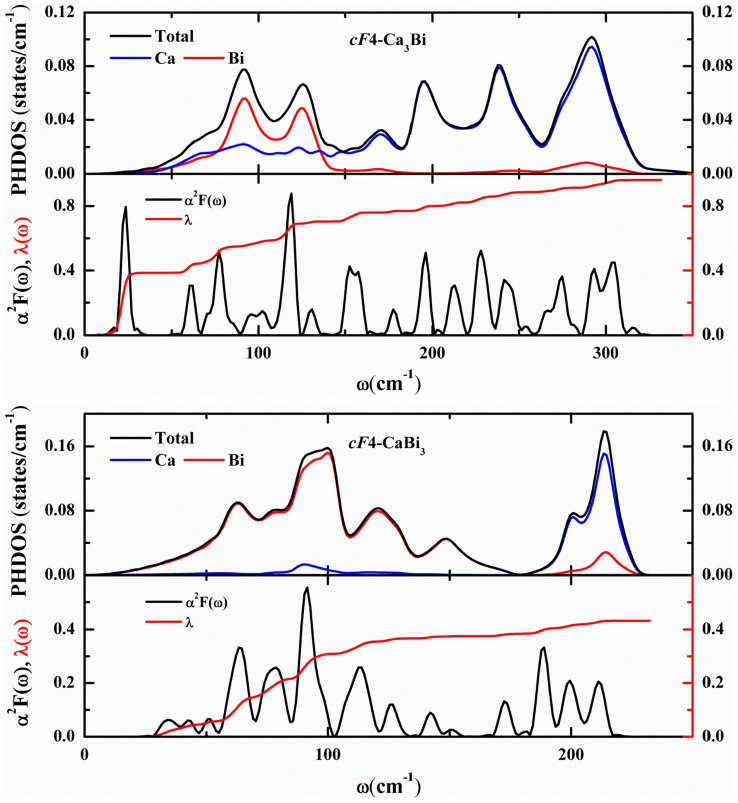
Total and projected phonon density of states (PHDOS) and Eliashberg function *α*^2^*F*(*ω*) for *cF*4-Ca_3_Bi at 60 GPa and *cF*4-CaBi_3_ at 30 GPa and the corresponding integrated electron-phonon coupling constant *λ*(*ω*).

The superconductivity of the selected structures can be conveniently studied by EPC calculation. The calculated Eliashberg spectral function and the electron-phonon coupling strength *λ* are shown in Fig. 5 for *cF*4-Ca_3_Bi and *cF*4-CaBi_3_ at 60 and 30 GPa, respectively. The superconducting critical temperature can be estimated from the Allen-Dynes modified McMillan equation^34^
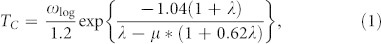
where the electron-phonon coupling constant is calculated as
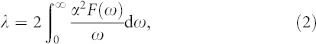
the logarithmic frequency average is
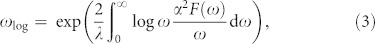
and a typical value of the Coulomb pseudopotential μ* = 0.10 is used.

**Figure 6:**
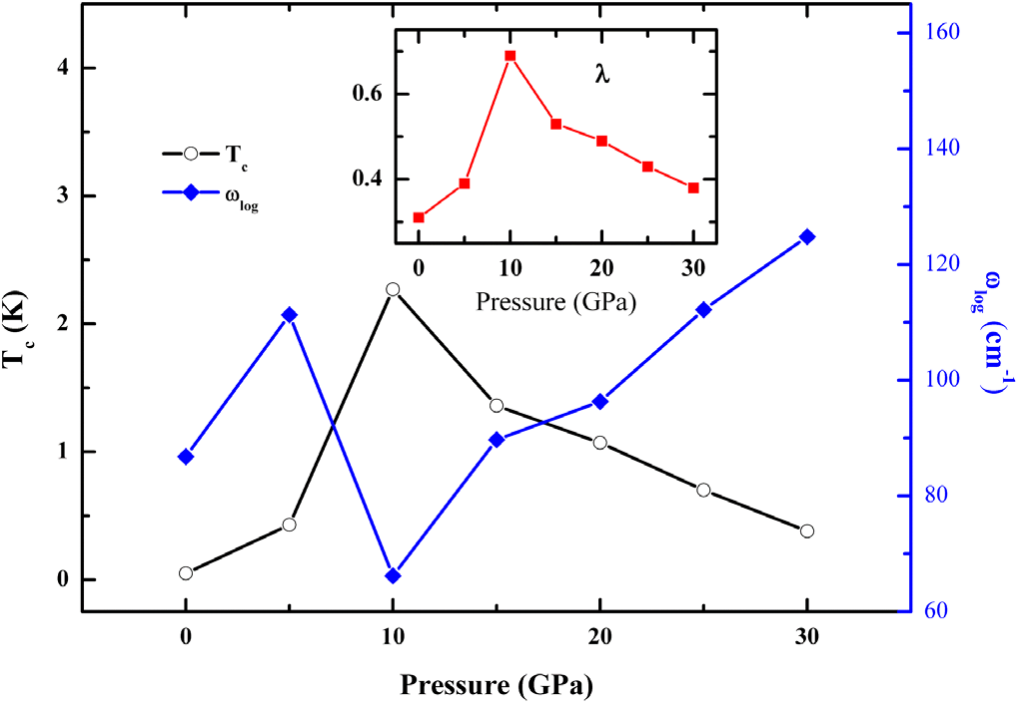
The calculated logarithmic average phonon frequency (*ω*_log_), EPC (*λ*) and critical temperature *T_c_* for *tI*2-CaBi as a function of pressure.

Our calculations suggest that *cF*4-Ca_3_Bi shows no superconductivity behavior below 25 GPa. Its superconducting transition temperature rises as the pressure increases. The dynamic instability of *cF*4-Ca_3_Bi is confirmed as the phonon spectrum displays imaginary frequencies above 60 GPa, at which we predict that *cF*4-Ca_3_Bi will be superconductor with a *T_c_* of 5.25 K, total *λ* of 0.96 and *ω*_log_ of 146.1 cm^−1^. 72% of the total *λ* results from modes below 140 cm^−1^, which are mainly displacements of bismuth atoms. The decomposition of the phonon density of states into contributions from the atoms (Fig. 5a) shows that the calcium atoms (below 140 cm^−1^) has a slight contribution to the electron-phonon coupling in this compound. On the contrary, *cF*4-CaBi_3_ can be a superconductor at 0 GPa with a *T_c_* of 5.16 K, total *λ* of 1.23 and *ω*_log_ of 56.2 cm^−1^, which will accordingly turn into 0.61 K, 0.41 and 127.8 cm^−1^ at 30 GPa. 84% of the total *λ* results from modes below 125 cm^−1^, which are mainly displacements of bismuth atoms, the same with *cF*4-Ca_3_Bi. A further study of the phonon density of states of *cF*4-CaBi_3_ indicates that the calcium atoms (below 180 cm^−1^) has a negligible contribution to the electron-phonon coupling in this compound (Fig. 5b). The calculated *λ*, *ω*_log_ and *T_c_* at different pressure for *cF*4-Ca_3_Bi and *cF*4-CaBi_3_ are listed in Table 2 and Table 3, respectively. The *tI*2-CaBi exhibits different superconductivity behavior through a brief study of our calculation as the *T_c_* will reach a maximum value of 2.27 K at around 10 GPa, as is shown in Fig. 6. A further analysis of the result shows that bismuth phonon mode is thought to play a large role in the superconductivity of *cF*4-Ca_3_Bi and *cF*4-CaBi_3_.

## Discussion

In summary, by using the variable-composition evolutionary algorithm, we performed a systematic search for all possible compositions in the Ca-Bi system at different pressures. Except the previously reported *oP*32-Ca_5_Bi_3_ and *tI*84-Ca_11_Bi_10_, we found 10 novel structures either totally unreported or only mentioned but no detail information. In addition, we predicted a series of phase transitions in Ca_2_Bi, Ca_3_Bi_2_ and CaBi, and also one stoichiometry (Ca_3_Bi) with a *cF*4 structure. All the newly predicted structures can be both dynamically and thermodynamically stable as the pressure increases. Based on conventional BCS theory, *cF*4-CaBi_3_ is superconductor with a *T_c_* of 5.16 K at 0 GPa and will drop with pressure increases. While *cF*4-Ca_3_Bi shows no superconductive behavior below 25 GPa and the *T_c_* value is enhanced with increasing pressure and reaches 5.25 K at 60 GPa. Compared to the above, *tI*2-CaBi is much different as the *T_c_* will reach a maximum value of 2.27 K at around 10 GPa. The newly predicted structures of calcium bismuthides and superconductivity behavior of *cF*4-CaBi_3_, *tI*2-CaBi and *cF*4-Ca_3_Bi would stimulate further experimental and theoretical studies on alkaline earth metal bismuthides and pnictide.

## Methods

We used the evolutionary algorithm USPEX to search for low-enthalpy stable structures as implemented in the USPEX code^35^,^36^, which has been widely used to predict stable high-pressure crystal structures without requiring any experimental information.

The underlying structural relaxations and electronic structure calculations of Ca-Bi over a wide range of the pressure presented here were performed within the density functional theory (DFT), using the all electron projector augmented wave (PAW) method^37^ as implemented in the Vienna *ab initio* simulation package (VASP)^38^. The 3s^2^3p^6^4s^2^ and 5d^10^6s^2^6p^3^ electrons are treated as valence electrons for Ca and Bi atoms, respectively. The exchange-correlation energy was treated within the generalized gradient approximation (GGA), using the functional of Perdew-Burke-Ernzerhof^39^ for both Ca and Bi. A plane-wave cutoff energy of 500 eV and dense Monkhorst-Pack *k*-point meshes^40^ with the reciprocal space resolution of 2π × 0.03 Å^−1^ were used for all structures to ensure that the enthalpy calculations are converged to better than 1 meV/atom.

The calculation of electron-phonon coupling (EPC) parameter *λ* are performed using the pseudopotential plane-wave method within the density functional perturbation theory (DFPT) as implemented in the Quantum Espresso package^41^ using Martins Troullier-type norm-conserving pseudopotential with cutoff energies of 80 and 360 Ry for the wave functions and the charge density, respectively. In order to interpolate the interatomic force constant matrix for the phonon dispersions, 4 × 4 × 4, 4 × 4 × 4 and 4 × 4 × 3 *q*-meshes in the first Brillouin zone (BZ) were used for interpolation for *cF*4-CaBi_3_, *cF*4-Ca_3_Bi and *tI*2-CaBi, respectively. The denser 24 × 24 × 24, 24 × 24 × 24 and 24 × 24 × 18 grids were sufficient to ensure the convergence needed for the EPC calculations for the three calcium bismuthides, respectively.

To ensure the dynamical stability of the newly predicted structures, the phonon dispersion curves were calculated throughout the Brillouin zone using the finite-displacement approach as implemented in the PHONOPY code^42^.

## Author Contributions

C.Z.F. conceived the idea. X.D. performed the *ab initio* evolutionary simulations and the superconductivity properties calculations. C.Z.F.and X.D. wrote the manuscript.

## Supplementary Material

Supplementary Information Supplementary Information

## Figures and Tables

**Table 1:** Crystal parameters for the predicted structures

Compound	Space group Pearson symbol	Lattice constants (Å)	Atom position (Wyckoff position)
Ca_2_Bi	*I*4/*mmm tI*12	*a* = 4.851 Å, *c* = 17.005 Å	Ca (4*c*) (0, 0.5, 0); (4*e*) (0, 0, 0.3255)
			Bi (4*e*) (0, 0, 0.1358)
	*Cmmm oI*12	*a* = 5.136 Å, *b* = 16.496 Å, *c* = 4.572 Å	Ca (4*i*) (0.5, 0.8224, 0); (2*a*) (0, 0, 0) (2*c*) (0.5, 0, 0.5)
			Bi (3*c*) (0.5, 0.5, 0)
	*P*6_3_/*mmc hP*6	*a* = 5.618 Å, *c* = 6.98 Å	Ca (2*d*) (0.3333, 0.6667, 0.75); (2*a*) (0, 0, 0.5)
			Bi (2*c*) (0.6667, 0.3333, 0.75)
Ca_3_Bi_2_	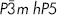	*a* = 5.304 Å, *c* = 7.212 Å	Ca (2*d*) (0.3333, 0.6667, 0.6702); (1*a*) (0, 0, 0)
			Bi (4*e*) (0.3333, 0.6667, 0.213)
	*C*2/*m mC*20	*a* = 17.239 Å, *b* = 4.894 Å, *c* = 8.112 Å,*β* = 101.329°	Ca (2*a*) (0.5, 0.5, 0); (4*i*) (0.6926, 0.5, 0.8385); (4*i*) (0.3506, 0, 0.5584); (2*c*) (0.5, 0.5, 0.5)
			Bi (4*i*) (0.3096, 0.5, 7598); (4*i*) (0.4428, 0, 0.2374)
	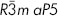	*a* = 5.3150 Å, *c* = 19.8239 Å,	Ca (3*a*) (0, 0, 0); (6*c*) (0, 0, 0.78103)
			Bi (6*c*) (0, 0, 0.40366)
CaBi_2_	*Cmcm oS*12	*a* = 4.782 Å, *b* = 17.160 Å, *c* = 4.598 Å	Ca (4*c*) (0, 0.9021, 0.25)
			Bi (4*c*) (0.5, 0.7578, 0.25); (4*c*) (0.5, 0.9339, 0.75)
Ca_3_Bi	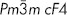	*a* = 5.108 Å	Ca (3*c*) (0.5, 0.5, 0)
			Bi (1*a*) (0, 0, 0)
CaBi	*P*4*/mmm tI*2	*a* = 3.786 Å, *c* = 4.355 Å	Ca (1*a*) (0, 0, 0)
			Bi (1*d*) (0.5, 0.5, 0.5)
	*I*4_1_*/amd tI*8	*a* = 4.628 Å, *c* = 11.656 Å	Ca (4*a*) (0.5, 0.25, 0.625)
			Bi (4*b*) (0, 0.25, 0.375)
CaBi_3_	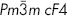	*a* = 4.998 Å	Ca (1*a*) (0, 0, 0) Bi (3*c*) (0, 0.5, 0.5)

**Table 2:** Calculated logarithmic average phonon frequency (*ω*_log_), EPC (*λ*) and critical temperature *T_c_* for *cF*4-Ca_3_Bi at selected pressures

Pressure(GPa)	*λ*	*ω*_log_(cm^−1^)	*T_c_*(K)
30	0.24	236.4	0.01
40	0.33	228.3	0.24
50	0.47	197.9	1.08
60	0.96	146.1	5.25

**Table 3:** Calculated logarithmic average phonon frequency (*ω*_log_), EPC (*λ*) and critical temperature *T_c_* for *cF*4-CaBi_3_ at selected pressures

Pressure(GPa)	*λ*	*ω*_log_(cm^−1^)	*T_c_*(K)
0	1.23	56.2	5.16
10	0.65	88.0	2.55
20	0.49	110.9	1.24
30	0.41	127.8	0.61
