# Supplementary material for: Rich stoichiometries of stable Ca-Bi system: Structure prediction and superconductivity
Source: Sci Rep. 2015 Mar 20;5:9326. doi: 10.1038/srep09326 (PMC4366814; doi:10.1038/srep09326)
Supplement: Supplementary Information [file srep09326-s1.pdf]

Supplementary information for

Rich stoichiometries of stable Ca-Bi system: Structure prediction and superconductivity

Xu Dong†, Changzeng Fan*†

†State Key Laboratory of Metastable Materials Science and Technology, Yanshan University, Qinhuangdao 066004, China

*For contact, E-mail: chzfan@ysu.edu.cn

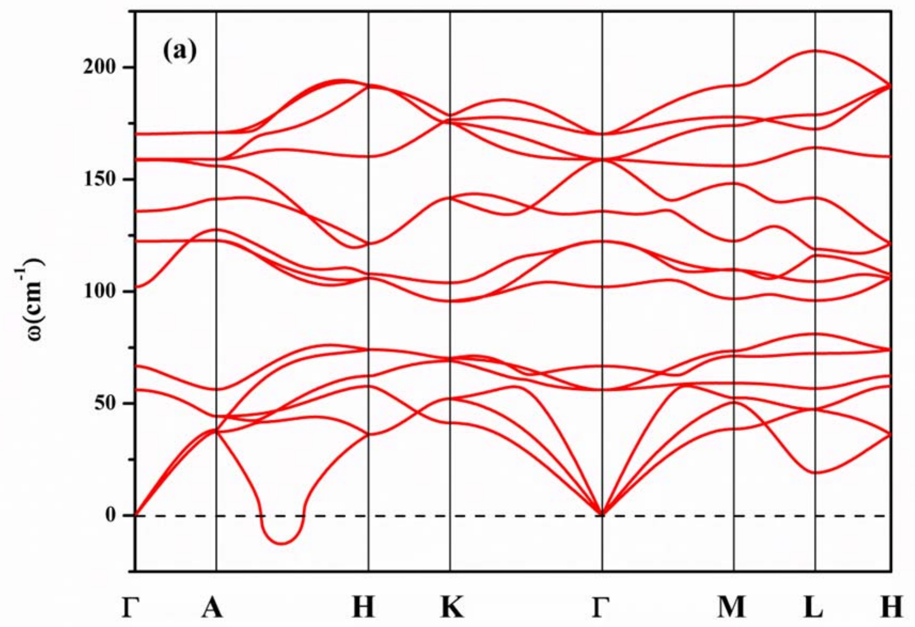
(a)
ω(cm^{-1})
200
150
100
50
0
Γ
A
H
K
Γ
M
L
H

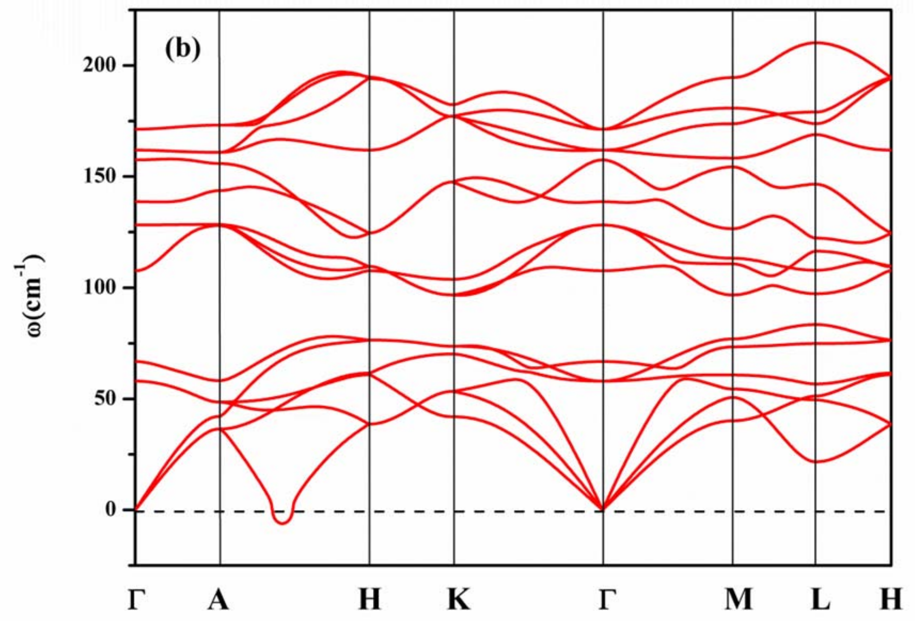
(b)
ω(cm^{-1})
200
150
100
50
0
Γ
A
H
K
Γ
M
L
H

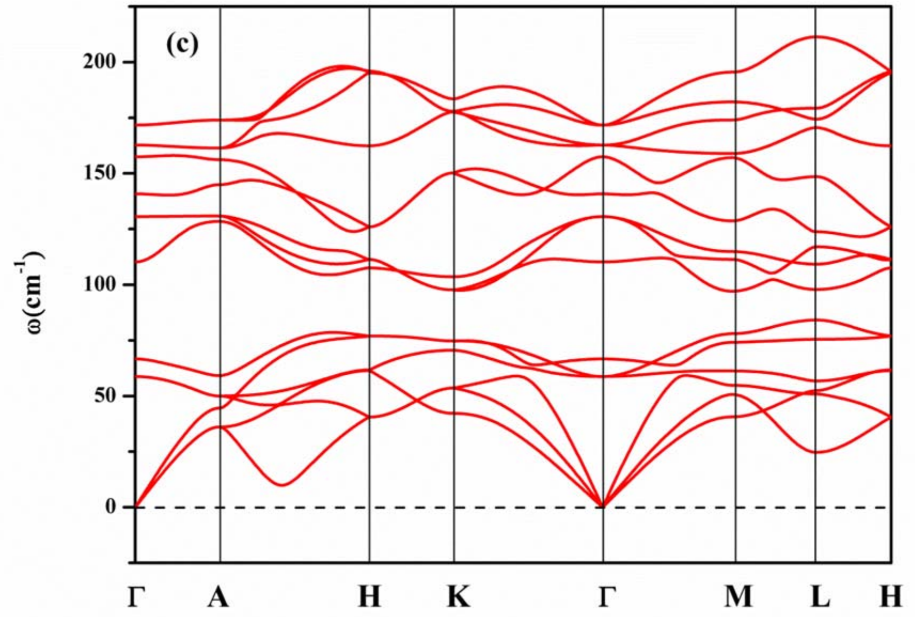

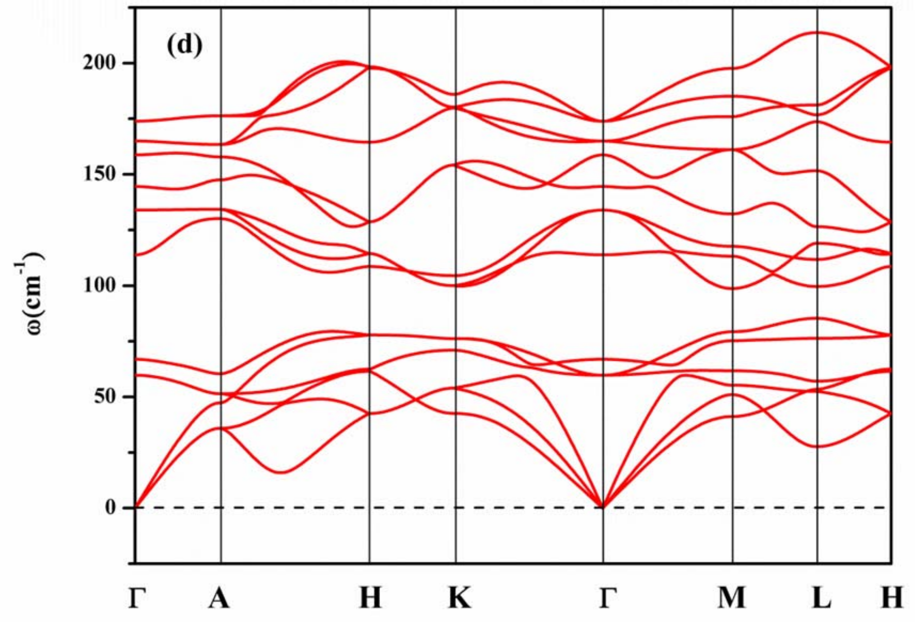

Figure S1. Phonon dispersion curves for the *hP*5-Ca_3Bi_2 from 0 GPa to 1 GPa. (a) 0 GPa, (b) 0.4 GPa, (c) 0.6 GPa, (d) 1 GPa.

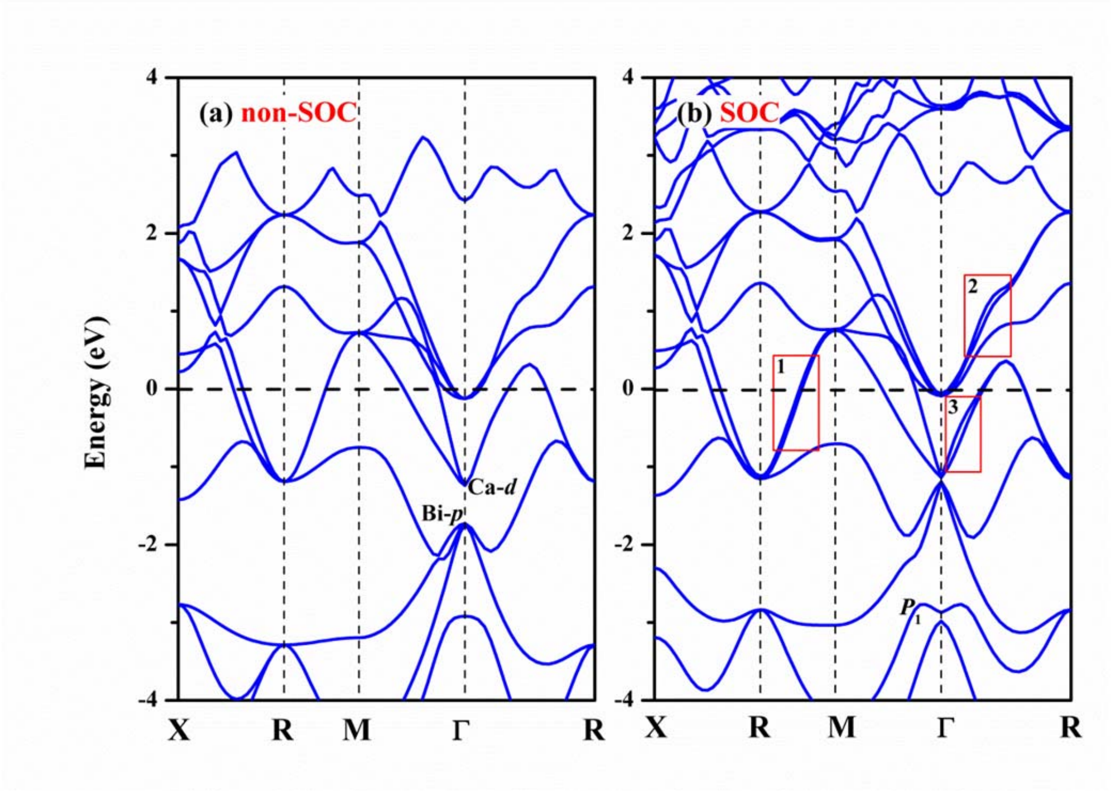

Figure S2. Band structures of *cF*4-Ca_3Bi. (a) and (b) are without and with SOC, respectively.

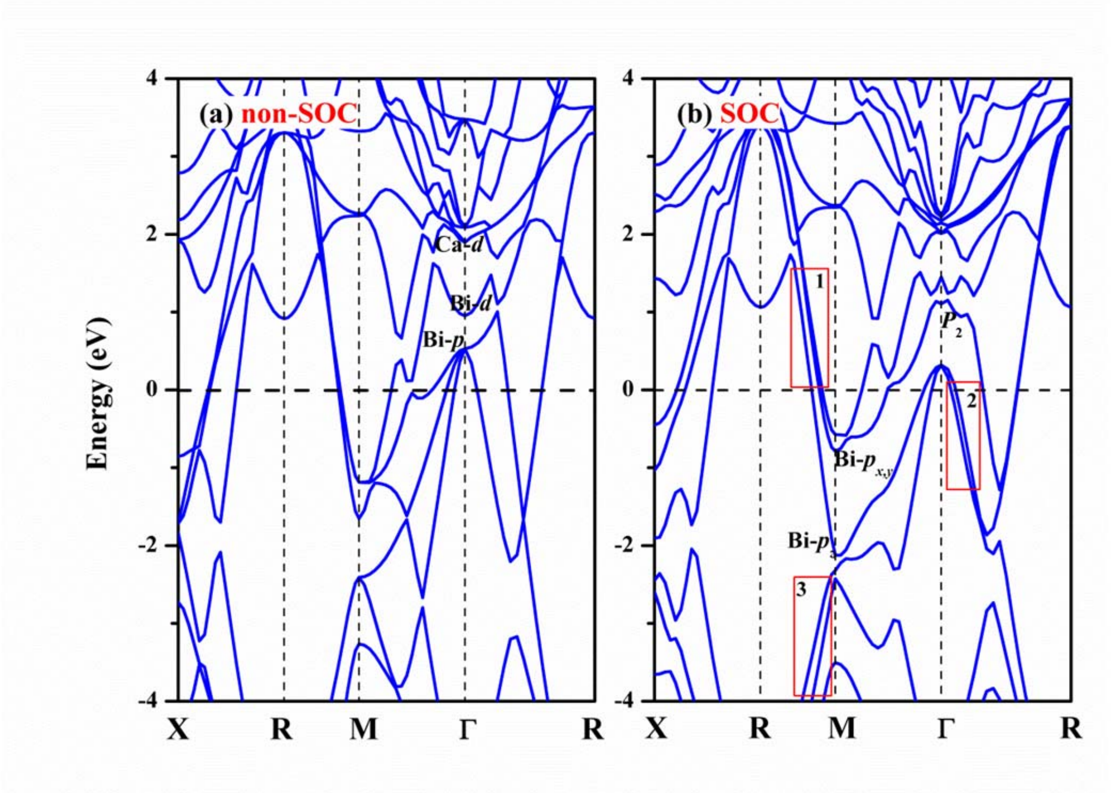

Figure S3. Band structures of *cF*4-$CaBi_3$. (a) and (b) are without and with SOC, respectively.

For $cF4$-Ca_3Bi, with the spin-orbit coupling (SOC) effect, the heavily overlapped bands don't separate each other much (as marked by 1-3 in Fig. S2 (a and b)). The most obvious is that, at Γ P_1 (as marked in Fig. S2(b)) states are split from the degenerated Bi-*p* orbitals. Besides, with the SOC effect, the gap between Ca-*d* orbitals and Bi-*p* orbitals will reduce from 0.48 eV to 0.08 eV.

For $cF4$-$CaBi_3$, the SOC effect results in the slight separations of the heavily overlapped bands (as marked by 1-3 in Fig. S3 (a and b)), which is similar to $cF4$-Ca_3Bi. At Γ, P_2 (as marked in Fig. S3(b)) states are split from the degenerated Bi-*p* orbitals, and lead to the lifting of Bi-*d* orbitals from 0.95 eV to 1.45 eV. The gap between Ca-*d* orbitals and Bi-*d* orbitals will accordingly reduce from 0.94 eV to 0.56 eV. Furthermore, under the SOC effect, at M the Bi-$p_{x,y}$ orbitals and Bi-p_z orbitals will be separated from 0.47 eV to 1.33 eV.
